# Bacterial Communities in the Womb During Healthy Pregnancy

**DOI:** 10.3389/fmicb.2018.02163

**Published:** 2018-09-06

**Authors:** Lihong Zhu, Fei Luo, Wenjing Hu, Yang Han, Yuezhu Wang, Huajun Zheng, Xiaokui Guo, Jinhong Qin

**Affiliations:** ^1^Department of Gynecology, Huadong Hospital, Fudan University, Shanghai, China; ^2^Department of Microbiology and Immunology, Institutes of Medical Sciences, Shanghai Jiao Tong University School of Medicine, Shanghai, China; ^3^Department of Microbiology, Guizhou Medical University, Guiyang, China; ^4^Shanghai-MOST Key Laboratory of Health and Disease Genomics, Chinese National Human Genome Center at Shanghai, Shanghai, China

**Keywords:** decidual tissue, amniotic fluid, V4 region of 16S rDNA, microbiome, proteobacteria, firmicutes

## Abstract

The idea that healthy uterine cavity is sterile is challenged nowadays. It is still debatable whether the bacteria present in the uterine cavity during pregnancy are residents or invaders. To reveal microbiome composition and its characteristics in the womb of pregnant women, 41 decidual tissue samples and 64 amniotic fluid samples were taken from pregnant Chinese women. DNA extraction was followed by pyrosequencing of the hypervariable V4 region of the 16S rDNA gene to characterize womb microbiome. Both types of samples had low diversity microbiome with Enterobacteriaceae being the dominant phylotypes at family level. To characterize the nature of colonization during pregnancy, the presence of endogenous biomass was confirmed by cultivation. Surprisingly, all of the 50 amniotic fluid samples studied were culture-negative, whereas 379 out of 1,832 placenta samples were culture-positive. Our results suggested that womb contained microbiome with low diversity. Culture-based investigation of amniotic fluid and placenta samples confirmed the presence of cultivable microorganisms in the placenta but not in amniotic fluid. Thus it suggests that bacterial colonization does occur during healthy pregnancy.

## Introduction

It was widely thought that the womb is a sterile environment during healthy pregnancy (Funkhouser and Bordenstein, 2013). The presence of bacteria in uterine cavity was considered as a risk factor because they could potentially affect the fetus and cause systemic inflammation and multiple organ damage (Martius and Eschenbach, 1990). The bacteria invading uterine cavity have been postulated to emerge mainly from the lower urogenital tract, ascending upward through the cervix to the uterus and then breaching the placental barrier to amniotic fluid and placenta (Goldenberg et al., 2000; Keelan and Payne, 2015). The presence of microorganisms such as *Ureaplasma* sp. and *Fusobacterium* sp. in uterine cavity confirmed by culture-dependent or culture-independent methods has been frequently associated with negative pregnancy outcomes (Han et al., 2004, 2009, 2010). However, despite the undoubtedly strong associations, these species have not been conclusively shown to be pathological agents. This circumstance indicates that available findings may have complex interpretation.

Recent advances in human microbiome investigations revealed an important role of microbes for human health (Human Microbiome Project Consortium, 2012). By using current sequencing technologies, multiple recent studies have challenged the traditional view of the womb as a sterile compartment. Amniotic fluid, the uterus, and the placenta, conventionally thought to be sterile, have recently been demonstrated to harbor unique microbiomes (Aagaard et al., 2014; Collado et al., 2016; Franasiak and Scott, 2017). We report here microbiomes of decidual tissue and amniotic fluid from healthy pregnancies elucidated by high-throughput sequencing technology and cultivable biomass of amniotic fluid and placenta by culture-based method.

## Materials and Methods

### Patient Recruitment and Ethical Considerations

Decidual tissue samples were obtained by curettage with vacuum aspiration from 41 pregnant women aged from 18 to 41 years with gestational age between 30 and 60 days. All cases were confirmed as normal intrauterine pregnancies by ultrasonography at the outpatient clinic of the Obstetrics and Gynecology Hospital before eligible artificial abortion. Women were excluded if: (1) they had been diagnosed with lower genital tract infections or other gynecological diseases; (2) had used any antimicrobials in the past 7 days; and (3) had used any vaginal devices or vaginally applied products in the past 30 days. The vulva, vagina, and cervix of the uterus were adequately sterilized before the operation. Specimens of decidual tissue were obtained from the participants after curettage with vacuum aspiration, and blood clots were removed. The samples were stored at -80°C for microbiome analysis.

Samples of amniotic fluid were collected from 64 Chinese women aged from 22 to 44 years with gestational age between 17 and 20 weeks in which the result of screening for Down’s syndrome in the fetus indicated high risk. Thus, amniocentesis for fetal karyotyping was recommended to these women at the Obstetrics and Gynecology Hospital. Women were excluded if: (1) they had been diagnosed with Down’s syndrome, lower genital tract infections, or other gynecological diseases; (2) had used any antimicrobials in the past 7 days; and (3) had used any vaginal devices or vaginally applied products in the past 17–20 weeks. After amniocentesis, amniotic fluid was centrifuged at 1,000 ×*g* to obtain cells for fetal karyotyping, and the remaining supernatant was further centrifuged at 10,000 ×*g* for microbiome analysis.

The clinical trial was registered with clinical trials (2017K055). All experiments were performed in accordance with guidelines and regulations of clinical trials (2017K055) approved by the Medical Ethics Committee of Huadong Hospital affiliated to Fudan University. All study participants gave their written informed consent for sample collection and subsequent microbiological analysis.

### Culturing Microbes From the Placenta and Amniotic Fluid

Amniotic fluid samples were collected from 50 pregnant women for medical diagnostics of aneuploidy as above. After centrifugation at 10,000 ×*g*, cells were plated onto brain heart infusion (Oxoid, Cambridge, United Kingdom) and Columbia blood agar plates (Oxoid, Cambridge, United Kingdom), which were aerobically incubated at 37°C for 48 h. A total of 1,832 placenta samples were collected by sterile swabs by using obstetrical standard operating procedures. Women aged 23–42 years were included if: (1) singleton gestation; (2) delivered at term (38^0/7^ to 41^6/7^ weeks gestational age); and (3) without clinical chorioamnionitis and other anomaly. Following standard obstetrical practices, after cesarean delivery of the infant and before the delivery of the placenta, swabs for microbial collection were swirled to fetal side to collect samples while taking care to avoid contamination from the maternal side. All the swabs were streak plated onto brain heart infusion (Oxoid, Cambridge, United Kingdom) and Columbia blood agar plates (Oxoid, Cambridge, United Kingdom), and cultivated aerobically at 37°C for 48 h. Conventional microbiological methods including analysis of colony and cellular morphology were used to preliminarily identify the isolates. Three clones each colony type was random selected to do Gram staining and observed. After preliminary identification, the same type clone was re-streaked to prepare a pure culture. Identification of viable organisms was carried out using VITEK 2 compact system (BioMerieux Inc., Marcy l’Etoil, France). GP, GN, NH, YST, and ANC cards were used.

### DNA Isolation and 16S rDNA Sequencing

Total genomic DNA of decidual tissue and amniotic fluid samples was extracted with a Power Soil DNA isolation Kit (cat. no. G-3246-50; Mo Bio Laboratories Inc., Carlsbad, CA, United States) according to manufacturer’s instructions. The quantity and quality of extracted DNA of each isolate was measured by PCR amplified with V3V5 primer (357F: 5′-CCTACGGGAGGCAGCAG-3′, 926R: 5′-CCGTCAATTCMTTTRAGT-3′). The amplification products were measured using a NanoDrop ND-1000 spectrophotometer (Thermo Fisher Scientific, Waltham, MA, United States) and agarose gel electrophoresis. When the amplicons were <500 ng, the extraction were not used as appropriate for sequencing. For sequencing, PCR amplification of hypervariable V4 regions of bacterial 16S rDNA genes was performed using forward primer 520F (5′-AYTGGGYDTAAAGNG-3′) and reverse primer 802R (5′-TACNVGGGTATCTAATCC-3′). Sample-specific 7-bp barcodes were incorporated into the primers for multiplex sequencing. After purification, PCR amplicons were pooled in equal amounts and pair-end 2 bp × 300 bp sequencing was performed using the Illlumina MiSeq platform with a MiSeq Reagent Kit v3 at Shanghai Personal Biotechnology Co., Ltd. (Shanghai, China).

The Quantitative Insights Into Microbial Ecology (QIIME, v. 1.8.0) pipeline was employed to process sequencing data as previously described (Caporaso et al., 2010). Briefly, raw sequencing reads with exact matches to the barcodes were assigned to respective samples and identified as valid sequences. The low-quality sequences, namely sequences that had a length of <150 bp, sequences that had average Phred scores of <20, sequences that contained ambiguous bases, and sequences that contained mononucleotide repeats of >8 bp were filtered out. Paired-end reads were assembled using FLASH (Magoc and Salzberg, 2011). After chimera detection, bacterial operation taxonomic units (OTUs) were clustered by UCLUST (QIIME^1^) based on 97% nucleotide similarity. A representative sequence selected from each OTU using default parameters. OTU taxonomic classification was conducted by using BLAST comparisons of the representative sequences set against the Greengenes Database using best hits. An OTU table was further generated to record the abundance of each OTU in each sample and taxonomy of these OTUs. OTUs containing <0.001% of total sequences across all samples were discarded. To minimize the difference of sequencing depth across the samples, an averaged, rounded, and rarefied OTU table was generated by averaging 100 evenly resampled OTU subsets under 90% of the minimal sequencing depth for further analysis.

### Bioinformatics and Statistical Analysis

Bioinformatics and statistical analyses of sequencing data were mainly performed using QIIME and R packages (v. 3.2.0). Relative abundance profiles at taxa levels (phylum, class, order, family, and genus) were generated based on OTU annotation. OTU-level alpha diversity indices such as Shannon diversity index were calculated using the OTU table in QIIME and plotted by GraphPad Prism 5 (GraphPad Software, Inc., San Diego, CA, United States). Beta diversity analysis was performed to investigate the structural variation of microbial communities across the samples using UniFrac distance metrics and visualized via non-metric multidimensional scaling (NMDS). Taxon abundances at the family and genus levels were statistically compared among the samples and plotted by GraphPad Prism 5.

## Results

### Diversity and Composition of Bacterial Communities in Decidual Tissues and Amniotic Fluids

Total DNA was isolated from collected samples, and the V4 region of the 16S rDNA gene was amplified and sequenced. A total of 2,401,802 filtered, high-quality sequences were produced in this study, with an average of 22,658 reads per sample. The validation sequences were clustered into 740 OTUs. All decidual tissue sample OTUs (100% of phylotypes) could be annotated at the phylogenetic level, as well as on family (99.9% of phylotypes) and genus levels (92.082% of phylotypes). Nearly all OTUs in amniotic fluid samples (99.976% of phylotypes) were also annotated at the phylogenetic level, including 97.823% of phylotypes at the family level and 54.091% of phylotypes at the genus level.

**FIGURE 1:**
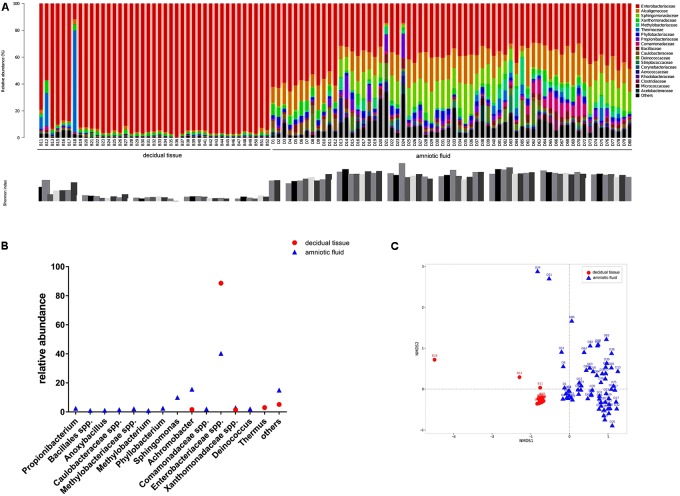
Bacterial profiling and diversity in each sample analyzed by 16S rDNA gene pyrosequencing. **(A)** Bacterial profiling plot of relative abundances of operational taxonomic units (OTUs) at the family level. Points plot of Estimators of the Shannon index are shown below. **(B)** Difference of bacterial taxa at the genus level between decidual tissue and amniotic fluid samples. **(C)** Non-metric multidimensional scaling of microbiome structure based on weighted UniFrac distance. Relative abundances of OTUs accounting for >0.1% of the total bacterial community are shown.

The diversity of microbes within a habitat was defined as the number and abundance distribution of distinct types of organisms. **Figure 1A** presents the relative abundances of OTUs at the family level in each sample along with the Shannon index. Microorganisms with relative abundance of >0.1% at the family level in two types of samples showed that Enterobacteriaceae accounted for 88.6% of the relative abundance in decidual tissue and for 40.3% in amniotic fluid. Detailed comparisons at the genus level across the two types of samples showed that Firmicutes were more abundant in decidual tissue than in amniotic fluid (**Figure 1B**) and 10 taxa, e.g., *Propionibacterium*, *Bacillales* spp., *Anoxybacillus*, *Caulobacteraceae* spp., *Methylobacteriaceae* spp., *Methylobacterium*, *Phyllobacterium*, *Sphingomonas*, *Comamonadaceae* spp., and *Deinococcus* were unique in amniotic fluid. Although Enterobacteriaceae were most abundant in most samples, differences in the compositions of bacterial communities were observed between decidual tissue and amniotic fluid. The Shannon indices indicated low diversity in both decidual tissue and amniotic fluid, although amniotic fluid diversity tended to be higher. When the community structures of decidual tissue and amniotic fluid were assessed by NMDS analysis of relative genus abundances, significantly separated clusters representing the two types of samples was demonstrated (**Figure 1C**).

### Recovery of Cultivable Biomass From Placental Samples but Not From Amniotic Fluid

In our studies, we further cultured 50 amniotic fluid samples and 1,832 placental samples. Out of 1,832 placental samples, 379 were culture-positive, whereas all 50 amniotic fluid samples were culture-negative in this study. In total, 447 strains of microorganisms were recovered from 1,832 placental samples (**Supplementary Table S1**). Of those, 428 were bacteria and 19 were fungi. All 19 fungi taxa were identified as belonging to the three species of the genus *Candida*: *Candida albicans*, *Candida tropicalis*, and *Candida glabrata*. Of the 428 recovered bacteria, 30 species were identified (**Table 1**). *Escherichia coli*, belonging to the family Enterobacteriaceae, accounted for 57.71% of the isolated strains, whereas *Enterococcus faecalis*, belonging to the family Enterococcaceae, accounted for 21.03% of the isolated strains.

**Table 1:** Bacteria recovered from the placenta under aerobic condition.

Strain	No.	Percentage
**Firmicutes**		
*Staphylococcus aureus*	8	1.87%
*Staphylococcus* sp.	8	1.87%
*Enterococcus faecium*	9	2.10%
*Enterococcus faecalis*	90	21.03%
*Enterococcus durans*	2	0.47%
*Enterococcus hirae*	1	0.23%
*Streptococcus salivarius*	1	0.23%
*Streptococcus pyogenes*	1	0.23%
*Streptococcus agalactiae*	22	5.14%
*Streptococcus bovis*	2	0.47%
*Streptococcus* sp.	2	0.47%
*Streptococcus pasteurianus*	1	0.23%
*Streptococcus mitis*	3	0.23%
*Listeria monocytogenes*	1	1.87%
**Proteobacteria**		
*Escherichia coli*	247	57.71%
*Klebsiella pneumoniae*	10	2.34%
*Klebsiella oxytoca*	1	0.23%
*Enterobacter aerogenes*	2	0.47%
*Proteus mirabilis*	1	0.23%
*Proteus vulgaris*	1	0.23%
*Proteus penneri*	1	0.23%
*Morganella morganii*	1	0.23%
*Citrobacter koseri*	3	0.70%
*Citrobacter braakii*	1	0.23%
*Citrobacter freundii*	3	0.70%
*Ewingella americana*	1	0.23%
*Raoultella planticola*	1	0.23%
*Aeromonas* sp.	1	0.23%
*Acinetobacter baumannii*	1	0.23%
*Sphingomonas* sp.	1	0.23%
**Actinobacteria**		
*Micrococcus* sp.	1	0.23%


## Discussion

The uterine cavity of healthy pregnant women has long been considered sterile, and it is traditionally believed that bacterial colonization of the womb is associated with adverse reproductive health outcomes, including miscarriage, chorioamnionitis, and preterm delivery. In recent years, although reports have shown that the womb is not a sterile environment, it is still debated if the microbiome detected in uterine cavity samples during healthy pregnancy represents real endogenous womb microflora or is a result of contamination (Jimenez et al., 2005, 2008; Stout et al., 2013; Aagaard et al., 2014; Wassenaar and Panigrahi, 2014; Lauder et al., 2016; Arora et al., 2017; Hornef and Penders, 2017). In our study, in order to avoid contamination, we obtained decidual tissue samples after curettage with vacuum aspiration. Our results showed that decidual tissue microbiome was low diversity and composed predominant phyla such as Proteobacteria, Thermus, and Firmicutes. *Enterobacteriaceae* sp. and *Thermus* were the dominant organisms at the genus level. Consistent with previous limited data on uterine microbiome composition from both culture-dependent and culture-independent assays, our experiments confirmed that uterine cavity harbored a limited number of particular phylotypes with low abundance and low diversity (Verstraelen et al., 2016; Walther-Antonio et al., 2016). However, our results showed that Proteobacteria taxa, such as Enterobacteriaceae, were the dominant organisms in the uterus of pregnant women, whereas previous reports indicated that Bacteroidetes were dominant organisms in the uterus of non-pregnant women (Verstraelen et al., 2016; Walther-Antonio et al., 2016).

Amniotic fluid samples in our study were collected in the middle trimester stage at the gestation age of 17 to 20 weeks by amniocentesis rather than at delivery in order to avoid contamination. We could recover microorganisms from the placenta by culture, but not from amniotic fluid. Thus, our results suggested that commensal and cultivable microbiota colonized the placenta. By using histological tissue staining, it has been previously demonstrated that the maternal basal plate of the placenta harbored microbes (Jimenez et al., 2005). Those data supported the idea that the placenta was not sterile. However, amniotic fluid samples in our study were culture-negative under both aerobic and anaerobic conditions. Recently, culture-based studies confirmed that amniotic fluid samples often (in >90% of cases) did not contain viable bacteria and only rarely were culture-positive (DiGiulio et al., 2010; Wassenaar and Panigrahi, 2014). In our study, although 16S rDNA sequencing detected microbiome in amniotic fluid, the samples were all culture-negative. It is possible that commensal microbiota in amniotic fluid has low abundance and thus, can only occasionally be isolated by culture, as noted by others. Another explanation is that commensal microbiota in amniotic fluid is adapted to specific environmental conditions and resists cultivation (a phenomenon called “the great plate count anomaly”) as suggested previously (Staley and Konopka, 1985; Romero et al., 2008). Finally, it is possible that healthy amniotic fluid indeed did not contain viable bacteria, and the detected microbiome actually is DNA release of microorganisms originating from other sites, such as from blood or placenta.

In summary, we present here a high-throughput assessment of womb microbiome. Microbiome structure of amniotic fluid was more diverse than that of decidual tissue, which supported the previous reports that bacteria could be hematogenously spread from blood to amniotic cavity (Aagaard et al., 2014). Thus, possibly, during healthy pregnancy, bacterial colonization occurs, however, chronological and mechanistic aspects of this phenomenon remain to be elucidated.

## Author Contributions

JQ and XG conceived the study. LZ, FL, WH, and YH performed the experiments. YW and HZ analyzed sequencing data. All authors contributed to the writing of the manuscript.

## Conflict of Interest Statement

The authors declare that the research was conducted in the absence of any commercial or financial relationships that could be construed as a potential conflict of interest.

## Footnotes

1 http://qiime.org/scripts/pick_otus.html

## Supplementary Material

TABLE S1 Detailed information of cultured organism from placenta.
